# EXPLORING THE IMPACT OF TECHNOLOGY ON OLDER ADULTS' WELL-BEING

**DOI:** 10.1093/geroni/igz038.696

**Published:** 2019-11-08

**Authors:** Walter R Boot

**Affiliations:** Florida State University, Tallahassee, Florida, United States

## Abstract

Recent research had indicated clear links between social isolation and loneliness, and a host of negative consequences, including poor mental and physical health, cognitive decline, and even increased mortality risk. These consequences make clear that social isolation is a significant and urgent public health concern that requires attention and action. Increasingly, researchers have begun to explore the role technology can play in reducing social isolation and increasing social support. This symposium contains five talks that address this question directly, presenting a nuanced picture of the potential effects of technology on well-being among older adults. The first presentation will highlight the potential positive side of internet use on wellbeing using longitudinal data from the Health and Retirement Study. The next talk explores how the internet can support older adults undergoing significant life transitions. Importantly, the link between internet use and well-being appears to be context-dependent, with internet use being associated with positive or negative effects depending on other contextual factors. This presentation is followed by another, finding that technology use can have varying effects depending on how and what the technology is used for. This is followed by a presentation that directly compares technology-based communication to in-person communication, and the different effects of each on well-being. The session concludes with a presentation on how novel robotic technology might provide emotional and social support. In answer to the question of whether technology can reduce social isolation and loneliness, and improve social support: It’s complicated.

